# Comparing Real and ChatGPT-Generated Radiographs for Training Deep Learning Models to Diagnose Knee Osteoarthritis

**DOI:** 10.7759/cureus.100381

**Published:** 2025-12-29

**Authors:** Rohan R Datir, Akshay Reddy, Yash Bhatia, Niall Baig, Roman Barrozo, Vinayak Sathe

**Affiliations:** 1 Medicine, California University of Science and Medicine, Colton, USA; 2 Medicine, Des Moines University, Des Moines, USA; 3 Medicine, University of Connecticut, Farmington, USA

**Keywords:** artificial intelligence, deep learning, osteoarthritis, radiograph analysis, synthetic imaging

## Abstract

Introduction: Osteoarthritis (OA) is a degenerative joint disease characterized by progressive cartilage loss, bone remodeling, and chronic pain. The growing global burden of OA motivates the evaluation of artificial intelligence (AI) approaches for automating radiographic diagnosis.

Purpose: This study aimed to compare AI models trained on real radiographs, ChatGPT-generated radiographs, and a combined dataset to assess whether synthetic imaging can improve OA detection.

Methods: Three binary classifiers were trained using knee radiographs: Model A (ChatGPT-generated images only), Model B (real images only), and Model C (real + synthetic). All models were developed using PyTorch in Google Colab and evaluated on 1,656 held-out real radiographs. Performance metrics (accuracy, sensitivity, specificity, precision, F1 score, and AUROC (area under the receiver operating characteristic)) were computed. Between-model comparisons used two-sided McNemar’s tests on paired predictions; 95% confidence intervals were estimated by bootstrap resampling. Grade-specific comparisons were Holm-Bonferroni adjusted (with unadjusted p-values also reported).

Results: Models B and C outperformed Model A across overall performance, while Model A showed higher specificity. Model C demonstrated slightly higher discrimination than Model B (AUROC 0.782 vs 0.758), with overlapping 95% confidence intervals. Sensitivity for grade 1 and grade 4 OA was higher for Model C than for Model B in unadjusted comparisons, but these differences did not remain statistically significant after Holm-Bonferroni adjustment.

Conclusion: ChatGPT-generated radiographs alone were insufficient for reliable training of OA diagnostic models. When used as a supplement to real radiographs, synthetic images produced small, directionally favorable changes in discrimination and grade-specific sensitivity, supporting their use as an adjunct for dataset expansion rather than a replacement for clinical imaging.

## Introduction

Osteoarthritis (OA) is a chronic, degenerative joint disorder marked by progressive loss of articular cartilage, subchondral bone remodeling, and synovial inflammation [1]. The disease arises from a complex interaction between mechanical stress and biochemical responses, which disrupt the equilibrium between cartilage synthesis and degradation [2]. Chondrocytes exposed to inflammatory cytokines such as interleukin-1 and tumor necrosis factor-alpha increase the production of matrix metalloproteinases, accelerating cartilage breakdown and altering joint architecture. Over time, this process leads to joint space narrowing, osteophyte formation, sclerosis, and pain, which are hallmark radiographic features of OA [1].

The severity of OA is commonly graded using the Kellgren-Lawrence (K-L) system, a standardized radiographic classification that ranges from grade 0 to grade 4 [3]. Grade 0 represents a normal joint, while grade 1 indicates doubtful osteophytes, grade 2 denotes definite osteophytes and possible joint space narrowing, grade 3 includes moderate osteophytes and sclerosis, and grade 4 shows severe deformity and large osteophytes. This scale is widely used to guide diagnosis, monitor disease progression, and evaluate treatment outcomes.

Globally, OA prevalence has more than doubled since 1990, rising from approximately 256 million to nearly 595 million cases in 2020, a 132% increase [4]. This trend has been attributed to population aging, obesity, and lifestyle factors that increase mechanical load on the joints [5]. High-income regions such as North America and Asia-Pacific report the greatest age-standardized prevalence, exceeding 8,000 cases per 100,000 individuals [4]. The growing disease burden highlights the need for scalable, objective diagnostic tools that can assist clinicians and improve early detection.

Artificial intelligence (AI), particularly deep learning (DL) and convolutional neural networks (CNNs), has shown promise in automating radiographic analysis of musculoskeletal diseases [6]. CNNs can identify complex image features such as osteophytes and joint space narrowing, potentially matching or exceeding expert-level interpretation accuracy [7]. However, a persistent limitation in medical AI development is access to sufficiently large and diverse datasets. For AI to generalize effectively, it must be trained on heterogeneous imaging data that represent a broad spectrum of disease severity and anatomical variation [8].

To address these limitations, recent advances in generative AI have enabled the creation of synthetic medical images that approximate real clinical data. ChatGPT’s image generation capabilities provide a practical, accessible means of producing such images from text prompts describing clinical or radiographic characteristics. Although generative adversarial networks (GANs) are traditionally used for synthetic imaging in radiology, ChatGPT offers an alternative approach that integrates text-based medical reasoning with visual generation. This capability may help expand training datasets in scenarios where obtaining real radiographs is restricted by privacy, cost, or availability.

Accordingly, this study investigates whether ChatGPT-generated radiographs can enhance AI model performance when diagnosing OA. Specifically, it compares three models: one trained exclusively on synthetic radiographs, one trained exclusively on real radiographs, and one trained on both combined. By analyzing sensitivity, specificity, accuracy, and F1 scores across disease grades, this work aims to determine whether ChatGPT-generated radiographs can serve as an effective supplement to real-world medical imaging in AI-based diagnosis.

## Materials and methods

Three AI models were developed and trained using knee radiographs to compare the diagnostic performance of real, synthetic, and combined datasets. The models were designated as Model A, Model B, and Model C. Each model consisted of two image classes: Healthy and Osteoarthritis. The datasets were organized in corresponding folders in Google Drive and processed using Google Colab.

A total of 1,526 de-identified knee radiographs (604 healthy and 922 osteoarthritic) were obtained from a publicly available Knee Osteoarthritis Severity Grading Dataset [8]. The dataset included Kellgren-Lawrence grade 0 (healthy) through grade 4 (severe OA) radiographs. The source dataset distribution was: grade 0 (n = 604), grade 1 (n = 275), grade 2 (n = 403), grade 3 (n = 200), and grade 4 (n = 44). Grade 0 images were assigned to the Healthy folder of Model B, while grades 1-4 images were assigned to the Osteoarthritis folder. For independent evaluation, an additional held-out test set of 1,656 real radiographs was used, with the following distribution: grade 0 (n = 639), grade 1 (n = 296), grade 2 (n = 447), grade 3 (n = 223), and grade 4 (n = 51).

The dataset was randomly split into training (90%) and validation (10%) subsets to evaluate model performance reproducibly. The same split ratio was maintained for all three models. To create the synthetic dataset, 604 grade 0, 275 grade 1, 403 grade 2, 200 grade 3, and 44 grade 4 radiographs were generated using ChatGPT’s image generation tool. These were produced based on text prompts requesting ChatGPT to generate radiographs of osteoarthritis of a particular grade, such as the following: “Create a batch of 36 x-rays of grade 1 osteoarthritis. Don’t include any arrows. Create some variation for each image to be distinct”. Synthetic knee radiographs were generated using OpenAI ChatGPT (model: GPT-5.2 Thinking) via the platform’s built-in image generation capability between June and August 2025. Images were produced using standardized prompts specifying Kellgren-Lawrence grade (0-4), AP knee radiograph appearance, and exclusion of annotations (e.g., “no arrows/text/labels”), with repeated generations until the target count per grade was reached. No explicit resolution or file-format parameters were set at generation time; images were downloaded using the platform’s default export settings. To support clinical plausibility, generated images were screened using a checklist aligned to Kellgren-Lawrence criteria; images with obvious non-anatomic artifacts (e.g., duplicated bony structures, implausible joint lines, distorted tibiofemoral alignment) were discarded (n = 0). All retained synthetic images underwent the same preprocessing pipeline as real radiographs (loading, resizing to the network input dimensions, normalization, and augmentation). These prompts were repeatedly requested to ChatGPT until the appropriate number of synthetic radiographs were generated for each grade. Each prompt was validated for clinical realism by referencing established Kellgren-Lawrence criteria [3]. The synthetic grade 0 images were stored in the Healthy folder of Model A, and the synthetic grade 1-4 images were stored in the Osteoarthritis folder.

Model C was constructed by merging the datasets used for Models A and B, combining both real and synthetic radiographs into shared Healthy and Osteoarthritis folders. This approach allowed assessment of whether synthetic data could improve model performance when used as a supplement to real imaging.

Each model was trained in Google Colab using PyTorch, employing a transfer learning approach with EfficientNet-B0, a widely used convolutional neural network architecture pre-trained on ImageNet. The model’s feature extraction layers were frozen, while the final classification block was fine-tuned for binary classification (Normal vs Osteoarthritis). Images were preprocessed with random rotation, horizontal flipping, and normalization to enhance generalization. The training parameters included a learning rate of 1×10⁻⁴, a batch size of six, and 100 training epochs with early stopping to prevent overfitting. Cross-entropy loss with label smoothing (0.1) and Adam optimization were applied, and the model with the best validation accuracy was saved as a .pth checkpoint.

For testing, 1,656 additional knee radiographs (639 healthy and 1,017 osteoarthritic) from the Knee Osteoarthritis Severity Grading Dataset [8] were used. Testing was conducted using Gradio, which generated a web interface that allowed drag-and-drop classification of radiographs. Each image’s diagnosis, Normal or Osteoarthritis, was recorded in a spreadsheet for analysis.

Model performance was assessed using accuracy, sensitivity, specificity, precision, and F1 score. Sensitivity was further stratified by grade (1-4). True positives, false negatives, false positives, and true negatives were computed for each model to generate confusion matrices. Because the same test images were evaluated by all models, model comparisons were treated as paired. Differences in classification error between two models were assessed using McNemar’s test on paired predictions. Uncertainty for performance metrics (accuracy, sensitivity, specificity, precision, F1 score, and AUROC (area under the receiver operating characteristic)) was quantified using nonparametric bootstrap resampling of the test set (10,000 resamples) to obtain 95% confidence intervals. AUROC and ROC curves were computed using the models’ predicted OA probabilities (recorded as “% OA” in the Gradio output). A two-sided p-value < 0.05 was considered statistically significant. Where multiple grade-specific comparisons were performed, p-values were adjusted using the Holm-Bonferroni method (with unadjusted p-values also reported). All analyses were performed using Python 3.10 and PyTorch 1.13 within Google Colab.

Although all models were trained as binary classifiers (Normal/Grade 0 vs Osteoarthritis/Grades 1-4), grade-stratified evaluation was performed by computing sensitivity separately for each Kellgren-Lawrence grade (e.g., grade 1 positives only) while using grade 0 radiographs as the negative class. This quantifies how well the binary classifier detects OA at specific severity levels without implying multiclass grade prediction.

## Results

**Figure 1:**
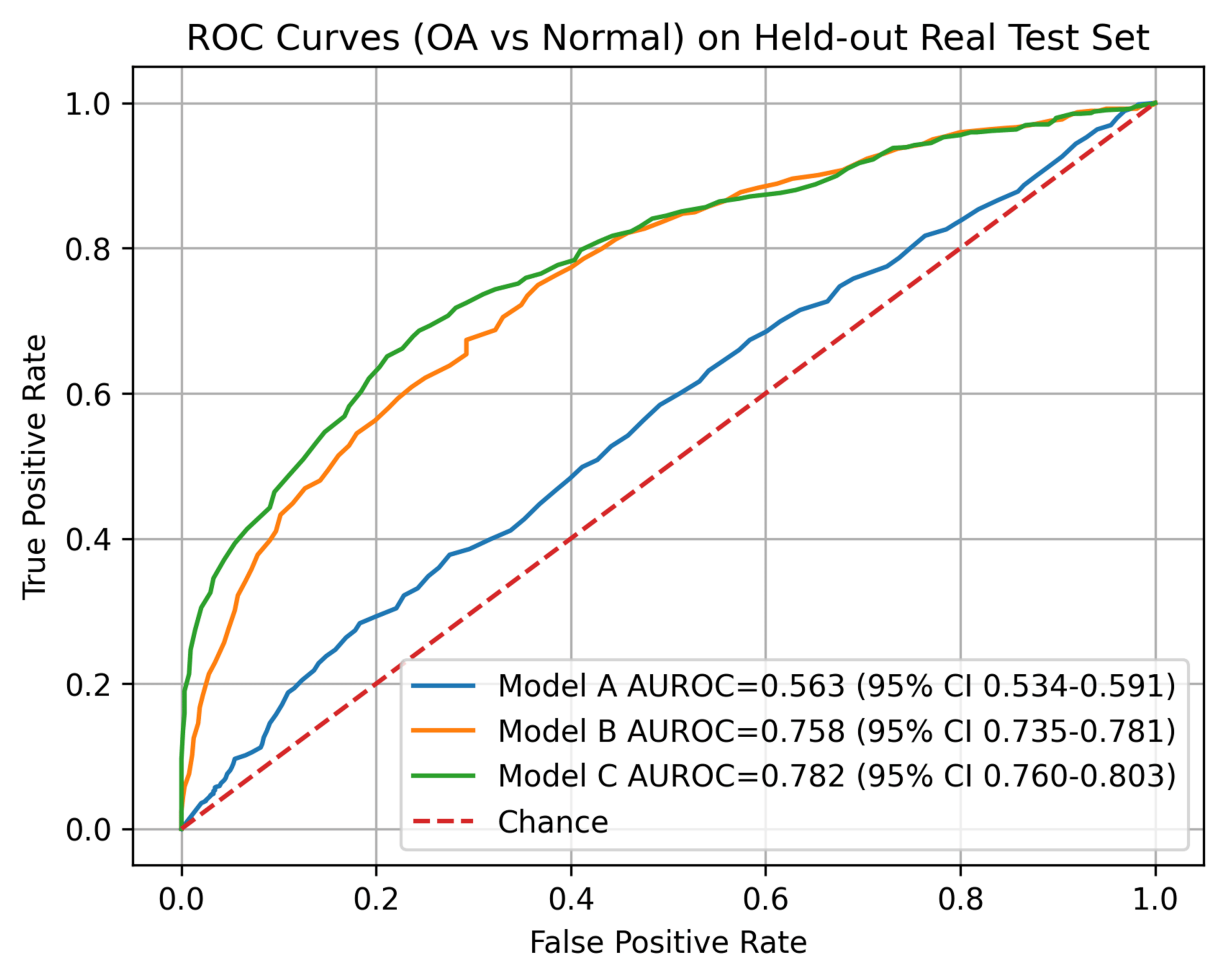
ROC curves for OA vs normal classification for Models A–C on the held-out real test set. AUROC values are shown with 95% bootstrap confidence intervals. ROC: receiver operating characteristic, OA: osteoarthritis, AUROC: area under the receiver operating characteristic curve, CI: confidence interval.

The performance of the three models, i.e., Model A (ChatGPT-generated radiographs), Model B (real radiographs), and Model C (combined datasets), was compared using confusion matrices, sensitivity analyses, and paired statistical tests. As shown in Table 1, each model produced different distributions of true positives, false negatives, false positives, and true negatives when evaluated on 1,656 real test radiographs, summarizing performance across osteoarthritis grades 1-4. Using the models’ predicted OA probabilities, AUROC on the held-out real test set was 0.563 (95% CI 0.534-0.591) for Model A, 0.758 (95% CI 0.735-0.781) for Model B, and 0.782 (95% CI 0.760-0.803) for Model C. ROC curves for Models A-C are shown in Figure 1 to compare discrimination across thresholds.

**Table 1:** True positives, true negatives, false positives, and false negatives for grades 1–4 for Model A, Model B, and Model C.

Model A Grades 1-4	Real Positive	Real Negative	Total
Predicted Positive	363	167	530
Predicted Negative	654	472	1091
Total	1017	639	1656

Model B Grades 1-4	Real Positive	Real Negative	Total
Predicted Positive	849	309	1158
Predicted Negative	168	330	498
Total	1017	639	1656

Model C Grades 1-4	Real Positive	Real Negative	Total
Predicted Positive	863	321	1184
Predicted Negative	154	318	472
Total	1017	639	1656

When examining individual disease grades, Tables 2-4 display the confusion matrices for each grade within the three models. Table 2 shows Model A’s grade-specific predictions, Table 3 illustrates Model B’s results, and Table 4 demonstrates Model C’s outcomes. As the tables show, Model A misclassified a greater number of mild (grade 1) and moderate (grade 2) OA cases as normal, indicating low sensitivity in early disease detection. In contrast, Models B and C demonstrated higher accuracy across all grades, particularly for grades 2-4, which represent more advanced OA. In the grade-stratified tables, the negative set (grade 0 radiographs) was held constant across grades; therefore, the false-positive count is the same across grade-specific tables within a given model by design, and grade-wise differences primarily reflect changes in true positives/false negatives (sensitivity).

**Table 2:** True positives, true negatives, false positives, and false negatives for grade 1, grade 2, grade 3, and grade 4 for Model A.

Grade 1	Real Positive	Real Negative	Total
Predicted Positive	100	167	267
Predicted Negative	196	472	668
Total	296	639	935

Grade 2	Real Positive	Real Negative	Total
Predicted Positive	143	167	310
Predicted Negative	304	472	776
Total	447	639	1086

Grade 3	Real Positive	Real Negative	Total
Predicted Positive	94	167	261
Predicted Negative	129	472	601
Total	223	639	862

Grade 4	Real Positive	Real Negative	Total
Predicted Positive	26	167	193
Predicted Negative	25	472	462
Total	51	639	690

**Table 3:** True positives, true negatives, false positives, and false negatives for grade 1, grade 2, grade 3, and grade 4 for Model B.

Grade 1	Real Positive	Real Negative	Total
Predicted Positive	206	309	515
Predicted Negative	90	330	420
Total	296	639	935

Grade 2	Real Positive	Real Negative	Total
Predicted Positive	400	309	709
Predicted Negative	47	330	377
Total	447	639	1086

Grade 3	Real Positive	Real Negative	Total
Predicted Positive	200	309	509
Predicted Negative	23	330	353
Total	223	639	862

Grade 4	Real Positive	Real Negative	Total
Predicted Positive	43	309	352
Predicted Negative	8	330	338
Total	51	639	690

**Table 4:** True positives, true negatives, false positives, and false negatives for grade 1, grade 2, grade 3, and grade 4 for Model C.

Grade 1	Real Positive	Real Negative	Total
Predicted Positive	227	321	548
Predicted Negative	69	318	387
Total	296	639	935

Grade 2	Real Positive	Real Negative	Total
Predicted Positive	388	321	709
Predicted Negative	59	318	377
Total	447	639	1086

Grade 3	Real Positive	Real Negative	Total
Predicted Positive	198	321	519
Predicted Negative	25	318	343
Total	223	639	862

Grade 4	Real Positive	Real Negative	Total
Predicted Positive	50	321	371
Predicted Negative	1	318	319
Total	51	639	690

Table 5 summarizes each model’s accuracy, overall and grade-specific sensitivity, specificity, precision, and F1 score. Model A, trained exclusively on ChatGPT-generated radiographs, achieved an overall accuracy below 0.7 and an F1 score under 0.5, indicating poor generalization to real-world data. Model B, trained solely on real radiographs, demonstrated substantially higher accuracy and F1 scores above 0.78, confirming the reliability of authentic imaging for AI training. Model C, which integrated both real and synthetic datasets, achieved the highest F1 score overall and slightly surpassed Model B in sensitivity for grades 1 and 4, suggesting a mild benefit of data augmentation with synthetic images.

**Table 5:** Accuracy, overall sensitivity, sensitivity stratified by grade, specificity, precision, and F1 score for Models A, B, and C.

Variables	Model A	Model B	Model C
Accuracy	0.50423	0.71196	0.71316
Sensitivity Overall	0.35693	0.83481	0.84857
Sensitivity Grade 1	0.33784	0.69595	0.76689
Sensitivity Grade 2	0.31991	0.89485	0.86801
Sensitivity Grade 3	0.42152	0.89686	0.88789
Sensitivity Grade 4	0.50980	0.84314	0.98039
Specificity	0.73865	0.51643	0.49765
Precision	0.68491	0.73316	0.72889
F1 Score	0.46929	0.78069	0.78419

Statistical comparisons between models were conducted using McNemar’s test to assess differences in paired classification error on the same test images. Table 6 summarizes McNemar p-values for A vs. B, B vs. C, and C vs. A. For grade-specific comparisons, Holm-Bonferroni adjusted p-values are reported alongside unadjusted p-values.

**Table 6:** McNemar’s test p-values for paired model comparisons on the same test images. Holm–Bonferroni–adjusted p-values are reported for grade-specific comparisons (grades 1–4 and grade 0) with unadjusted p-values shown alongside. Bold indicates Holm–Bonferroni–adjusted p < 0.05 for grade-specific comparisons. Unadjusted p-values are shown for reference.

Comparison	A vs. B (p)	A vs. B (Holm)	B vs. C (p)	B vs. C (Holm)	C vs. A (p)	C vs. A (Holm)
Grades 0-4	8.05E-33	N/A	1	N/A	4.44E-31	N/A
Grades 1-4	1.30E-108	N/A	0.335	N/A	5.54E-106	N/A
Grade 1	1.11E-19	3.33E-19	0.022	0.088	1.86E-25	7.45E-25
Grade 2	5.32E-66	2.66E-65	0.175	0.526	3.33E-55	1.66E-54
Grade 3	9.53E-26	3.81E-25	0.86	0.86	1.55E-22	4.66E-22
Grade 4	4.88E-04	4.88E-04	0.016	0.078	8.05E-07	8.05E-07
Grade 0	3.14E-17	6.28E-17	0.42	0.839	2.06E-19	4.12E-19

Collectively, these results demonstrate that while ChatGPT-generated radiographs alone are insufficient for reliable diagnostic training, supplementing real data with synthetic examples may modestly improve model sensitivity for both early and severe OA grades. No overlap was detected between patient data in the training and test sets, confirming dataset independence.

## Discussion

This investigation highlights the role of AI models in automating OA diagnosis and clarifies the contribution of ChatGPT-generated radiographs as supplementary data. The three models demonstrated markedly different diagnostic performance profiles, emphasizing that while synthetic images may expand training data, they cannot replace authentic radiographic inputs for reliable model training.

Models B and C, trained on real or combined datasets, outperformed Model A across nearly all metrics, including accuracy, sensitivity, precision, and F1 score, underscoring the necessity of real radiographs for model validity.

In addition, discrimination measured by AUROC was higher for Models B and C than for Model A when evaluated on real radiographs. Model C showed a modest AUROC advantage over Model B, although the 95% confidence intervals overlapped, suggesting that any improvement in overall discrimination was small in magnitude. Reporting AUROC with bootstrap confidence intervals and ROC curves provides a more complete view of uncertainty and threshold-dependent trade-offs, and external validation on multi-institutional datasets with different acquisition characteristics remains necessary to assess generalizability.

Notably, AUROC reflects the model’s ability to rank positive cases above negative cases across all decision thresholds, whereas accuracy depends on a single operating threshold. Therefore, it is possible to observe very high AUROC even when accuracy and specificity are not maximal at the chosen threshold. Future work could explore threshold optimization and calibration to better balance sensitivity and specificity depending on the intended clinical use case, such as screening versus confirmatory assessment.

The superior specificity observed in Model A suggests that models trained solely on synthetic data may develop a bias toward classifying ambiguous images as normal. This tendency likely arises because synthetic images, though visually plausible, lack the subtle pathological variation present in clinical radiographs. Such differences in textural detail and bone density representation may lead to under-detection of disease-positive cases.

Between Models B and C, differences were generally small. Model C showed higher sensitivity for grade 1 and grade 4 OA than Model B in unadjusted comparisons, but these differences did not remain statistically significant after Holm-Bonferroni adjustment (Table 6) and should be interpreted as directional. These findings suggest that while synthetic data may not globally improve performance, it can help address gaps at the extremes of disease severity, including cases that are either too subtle or too rare in real datasets. In this context, ChatGPT-generated radiographs appear to act as a form of data regularization by exposing the network to a broader spectrum of visual patterns that may enhance generalization. Importantly, because Model C was trained with a larger dataset than Models A and B, these gains cannot be attributed solely to the synthetic nature of the images. Instead, they highlight how data quantity and diversity jointly contribute to model robustness.

This study’s results align with prior research showing that increasing dataset size, whether through real or synthetic means, improves model generalization in medical imaging [9]. However, ChatGPT-based image generation represents a distinct approach from traditional methods such as generative adversarial networks (GANs). Unlike GANs, which require pre-existing imaging data for training, ChatGPT’s multimodal generation uses text-based prompts to create new, concept-driven radiographs. This characteristic makes it accessible to non-specialists and potentially useful in settings where obtaining real medical images is constrained by privacy or resource limitations.

Despite these advantages, the findings confirm that ChatGPT-generated images should be viewed as a supplement rather than a replacement for clinical data. Their role is best suited for augmenting underrepresented categories, such as early OA grades, where diagnostic features are subtle and often missed. The slight increase in Model C’s grade 1 sensitivity supports this interpretation. Additionally, the results suggest that hybrid datasets can improve F1 scores by balancing true positive and false positive rates, leading to more stable performance across classes.

This work also reinforces the principle that AI reproducibility depends on transparency in algorithm selection and dataset preparation. To ensure replicability, all models were developed in Google Colab using PyTorch and the EfficientNet-B0 architecture, a convolutional neural network known for computational efficiency and strong feature extraction performance. Synthetic knee radiographs were generated and augmented in alignment with the Kellgren-Lawrence grading framework to enhance clinical plausibility and task relevance for OA grading, addressing common concerns about unvalidated generative outputs in prior work [10].

Taken together, these results support the potential of multimodal AI systems such as ChatGPT to complement medical datasets by offering a low-cost approach to enhancing diagnostic AI development. However, the findings should be interpreted within the scope and limitations of the present study.

Future applications and limitations

Although this study systematically explores the influence of ChatGPT-generated radiographs on AI-based OA diagnosis, several limitations warrant consideration. First, the results are specific to the EfficientNet-B0 convolutional neural network architecture implemented in PyTorch within Google Colab. Performance outcomes may differ with alternative frameworks or architectures such as DenseNet, ResNet, or Vision Transformers, each of which extracts and weights radiographic features differently [11].

Second, the ChatGPT-generated dataset was created through text prompts describing each Kellgren-Lawrence grade. While prompts were informed by established diagnostic criteria and visually inspected for plausibility, they may not fully replicate the diversity and subtle artifact patterns present in real radiographs. The lack of inter-prompt variability could limit generalizability, and small errors in prompt wording may have amplified systematic bias in the generated images.

Third, Model C incorporated both synthetic and real radiographs, resulting in a larger overall training set. Although this configuration simulated a real-world dataset expansion, it also confounded the ability to isolate the unique effect of synthetic data. Future studies could address this limitation by creating a size-controlled model, for example, Model D, that equalizes image counts across training sets to directly measure the impact of synthetic versus real data alone.

Fourth, this investigation focused solely on knee radiographs. While the knee is the most frequently affected joint in OA, extension to other joints such as the hip or hand is essential to confirm generalizability. Similarly, only radiography was used as an imaging modality; incorporating magnetic resonance imaging (MRI) or computed tomography (CT) could help determine whether synthetic image supplementation provides consistent benefits across modalities.

Fifth, although the Kaggle dataset used here was publicly available and de-identified, it represents a single data source. Multi-institutional datasets would likely enhance diversity in image quality, scanner type, and population demographics, thereby improving model robustness [12].

Finally, the study did not compare ChatGPT-generated radiographs with those produced by other generative approaches, such as generative adversarial networks or diffusion models. Future research could systematically benchmark ChatGPT-based generation against established synthetic imaging frameworks to determine whether multimodal language models provide unique diagnostic advantages.

Future investigations may also explore hybrid systems that combine AI-generated images with human-in-the-loop feedback, allowing radiologists to iteratively refine the realism of synthetic images. Such work could help establish best practices for incorporating synthetic medical imaging into machine-learning pipelines while maintaining ethical and clinical validity.

## Conclusions

This study demonstrates that models trained exclusively on ChatGPT-generated radiographs generalize poorly to real knee radiographs, whereas models trained on real radiographs (with or without synthetic supplementation) achieve substantially stronger performance. Adding synthetic radiographs to real training data (Model C) produced modest improvements in overall discrimination (AUROC) and directionally higher sensitivity for early (grade 1) and severe (grade 4) OA compared with real-only training (Model B), although grade-specific differences were not statistically significant after Holm-Bonferroni adjustment. These findings suggest that synthetic images may be useful for augmenting training data, particularly where real data are limited or imbalanced, while not replacing clinical radiographs for model development and validation.

Future work should validate these findings on multi-institutional datasets, evaluate alternative architectures and calibration/threshold selection, and benchmark ChatGPT-generated images against established synthetic imaging methods to define best practices for incorporating multimodal synthetic data into clinical AI pipelines.
